# Obstetric and neonatal outcomes in women with pregnancy associated cancer: a population-based study in Lombardy, Northern Italy

**DOI:** 10.1186/s12884-020-03508-4

**Published:** 2021-01-07

**Authors:** Giovanna Esposito, Matteo Franchi, Michela Dalmartello, Giovanna Scarfone, Eva Negri, Fabio Parazzini, Carlo La Vecchia, Giovanni Corrao

**Affiliations:** 1 National Centre for Healthcare Research and Pharmacoepidemiology, Milan, Italy; 2 Laboratory of Healthcare Research & Pharmacoepidemiology, Department of Statistics and Quantitative Methods, University of Milano-Bicocca, Milan, Italy; 3 Department of Clinical Sciences and Community Health, University of Milan, 20122 Milan, Italy; 4 Department of Obstetrics, Gynecology and Neonatology, University of Milan, Fondazione IRCCS Ca’ Granda Ospedale Maggiore Policlinico, 20122 Milan, Italy

**Keywords:** Cancer, Pregnancy, Neonatal outcome, Pregnancy outcome, Population-based

## Abstract

**Background:**

Pregnancy associated cancer (PAC) may lead to adverse obstetric and neonatal outcomes. This study aims to assess the association between PACs and adverse perinatal outcomes [i.e. labor induction, iatrogenic delivery, preterm birth, small for gestational age (SGA) newborn, low Apgar score, major malformations, perinatal mortality] in Lombardy, Northern Italy.

**Methods:**

This population-based historic cohort study used the certificate of delivery assistance and the regional healthcare utilization databases of Lombardy Region to identify beneficiaries of National Health Service who delivered between 2008 and 2017. PACs were defined through oncological ICD-9-CM codes reported in the hospital discharge forms. Each woman with PAC was matched to four women randomly selected from those cancer-free (1:4). Log-binomial regression models were fitted to estimate crude and adjusted prevalence ratio (aPR) and the corresponding 95% confidence interval (CI) of each perinatal outcome among PAC and cancer-free women.

**Results:**

Out of the 657,968 deliveries, 831 PACs were identified (1.26 per 1000). PAC diagnosed during pregnancy was positively associated with labor induction or planned delivery (aPR=1.80, 95% CI: 1.57–2.07), cesarean section (aPR=1.78, 95% CI: 1.49–2.11) and premature birth (aPR=6.34, 95% CI: 4.59–8.75). No association with obstetric outcomes was found among PAC diagnosed in the post-pregnancy. No association of PAC, neither during pregnancy nor in post-pregnancy was found for SGA (aPR=0.71, 95% CI: 0.36–1.35 and aPR=1.04, 95% CI: 0.78–1.39, respectively), but newborn among PAC women had a lower birth weight (*p*-value< 0.001). Newborns of women with PAC diagnosed during pregnancy had a higher risk of borderline significance of a low Apgar score (aPR=2.65, 95% CI: 0.96–7.33) as compared to cancer-free women.

**Conclusion:**

PAC, especially when diagnosed during pregnancy, is associated with iatrogenic preterm delivery, compromising some neonatal heath indicators.

**Supplementary Information:**

The online version contains supplementary material available at 10.1186/s12884-020-03508-4.

## Background

Pregnancy associated cancer (PAC) is defined as cancer diagnosed during pregnancy or within 12 months following childbirth or abortion [1–5]. Its prevalence is reported to be about 1/1000 pregnancies [6]. A diagnosis of PAC may affect women’s treatment and survival, due to the challenge of targeting both maternal and fetal health. In addition, PAC, especially when diagnosed during pregnancy, may lead to adverse obstetric and neonatal outcomes [7]. About 15% of women with a diagnosis of PAC experience obstetric complications [i.e. premature membrane rupture prior to week 37 (PPROM), preeclampsia, blood loss and intrauterine growth restriction (IUGR)]. Nevertheless, the risk does not appear to be significantly higher than the general population [8]. Compared to healthy women, the risk of spontaneous preterm birth is also not significantly higher [9]. In contrast, medically induced labor or elective cesarean section are more frequently performed, due to the need of an early pregnancy termination in order to start surgical or pharmacological treatment [8–10]. Nevertheless, most of these results come from clinical series based on a specific context. For example, a recent observational cohort study [11] of women diagnosed with cancer in pregnancy aims to investigate the use of chemotherapy during the first trimester. Initiating chemotherapy in the first period of pregnancy significantly increase risk of IUGR [Odds Ratio (OR)=3.00], of congenital anomalies (OR=3.9) and of preterm birth (OR=2.3). Limited evidence has been published on the perinatal outcomes in women with PAC in population-based studies [6].

In order to add further evidence on obstetric and neonatal outcomes in women with and without PAC, a large retrospective population-based cohort study, including women who delivered between 2008 and 2017 in Lombardy, Northern Italy, was carried out.

## Methods

### Data sources

Data were retrieved from the regional healthcare utilization (HCU) databases of Lombardy Region, the most populated region in Northern Italy, accounting for about 10 million inhabitants (17% of the national population). All Italian citizens have equal access to health care services as part of the National Health Service (NHS), and in Lombardy this has been associated with an automated system of databases to collect a variety of information on residents who receive NHS assistance (NHS beneficiaries), diagnoses and procedures of inpatients of public or private hospitals (coded according to the ICD-CM-9 codes), outpatient drug prescriptions (coded according to the Anatomical Therapeutic Chemical (ATC) Classification System), specialist visits and diagnostic examinations reimbursable by the NHS. In addition, a database reporting the Certificates of Delivery Assistance (i.e., the so called CeDAP registry) providing detailed information on the mother’s socioeconomic traits, as well as detailed information regarding course of pregnancy, labor (e.g. induction), delivery (e.g. mode and timing), health of newborn (e.g. birth weight, Apgar score, presence of congenital malformation or major defect) and causes of perinatal mortality where applicable. A deterministic record linkage between databases through a unique identification code systematically used for all databases allows the identification of large and unselected birth cohorts and the possibility of establishing relevant traits and care pathways of mothers and newborns [12]. To preserve privacy, each identification code is automatically anonymized and the inverse process is allowed only to the Regional Health Authority on request from judicial authorities. Diagnostic, therapeutic and procedural codes used for the current study are given in Supplementary material (Table S1).

As previously underlines the analysis are based on anonymized data: no permission by Ethic Committees is necessary to collect and analyze these data.

### Study cohort and PAC definition

All the deliveries in Lombardy between 1st January 2008 and 30th June 2017 from women who (i) were beneficiaries of NHS and resident in Lombardy (at least since three years before the conception date and one year after the delivery date), (ii) were aged 12 to 55 years at delivery, (iii) had 22 to 42 weeks of gestation, and (iv) did not have an ICD-9 code related to cancer in the three years prior to the conception date were identified from CeDAP database. Among these, we excluded deliveries of newborns with very low birth weight (i.e., less than 400 g), deliveries with missing values on Apgar score, birth weight and vitality status, deliveries which did not match to a hospital ICD-9 code related to childbirth, and those in which the infant could not be linked to the mother because of a missing identification code.

Information on cancer diagnosis was obtained from the inpatient database. Accordingly, women with PAC were defined as those having an ICD-9 code of cancer, either in the main or in secondary diagnoses, during the period from conception to delivery (i.e., during pregnancy) or during the following 12 months (i.e. post-pregnancy). Cancer cases reported as secondary diagnosis, for which the main diagnosis was unrelated to cancer, were excluded.

Each woman with PAC was matched to four women randomly selected from those cancer-free, having the same maternal age and year of delivery. PAC and cancer-free women were also compared for socio-demographic characteristics (including nationality, marital status, education and employment), type of conception (i.e. spontaneous or assisted), multiple births, parity and previous history of type 2 diabetes and hypertension (detected in the three years before the date of conception).

### Outcomes of interest

Information on pregnancy, delivery and newborn were retrieved from the CeDAP database. Adverse obstetric outcomes included type of labor (induced or no labor due to elective cesarean section vs spontaneous), mode of delivery (cesarean section vs vaginal or instrumental) and timing of delivery (preterm, i.e. 37 gestation weeks or less, vs at term [13]). Adverse neonatal outcomes included small for gestational age (SGA) newborn, low Apgar score at 5 min (7 or less [14]), perinatal death and major malformations diagnosed before discharge of the newborn. SGA was defined as having a birth weight below the 10th percentile for gestational age, according to the sex-specific Italian reference curve for normal fetal growth [15].

### Statistical analyses

Descriptive statistics were used to summarize characteristics of PAC and cancer-free women. Differences on categorical variables between the two groups were tested by using absolute standardized differences. Numerical variables were compared between two groups by using the t-test for independent samples.

The overall prevalence of PAC in our study cohort was calculated by dividing the observed number of PAC by the total number of deliveries. Prevalence were further stratified by cancer site. Log-binomial regression models were fitted to estimate the prevalence ratio (PR) and the corresponding 95% confidence interval (CI) of each perinatal outcome among PAC and cancer-free women. Other than the crude PR, adjusted PR (aPR) were also estimated, with allowance for socio-demographic characteristics (nationality, marital status, education and employment), type of conception (i.e. spontaneous of assisted), multiple births, parity and previous history of type 2 diabetes and hypertension. Given the low number of observed cases for some of the outcomes considered, we could not adjust the model for all the aforementioned covariates. Thus, we first used a multivariate logistic regression model for estimating the probability of having a diagnosis of PAC, given the characteristics reported below, and then we used this probability as adjustment covariate in the log-binomial regression model [16]. Moreover, because of the potential correlation of women contributing to more than one birth during the considered period, the models were fitted using generalized estimating equations (GEE) for correlated observations with a log link [17]. Finally, because socio-demographic characteristics were missing for some women (< 3%), multiple imputations were applied by using the fully conditional specification (FCS) method, imputing 20 datasets [18, 19].

All the analyses were performed among strata of timing of PAC diagnosis (i.e. during pregnancy and post-pregnancy). The frequencies of perinatal outcomes were also reported separately for the three most common cancer sites (i.e. breast, thyroid cancer and lymphoma). Chi-squared test or Fisher’s exact test was used to evaluate differences between cancer sites.

All analyses were performed using the Statistical Analysis System Software (version 9.4; SAS Institute, Cary, NC, USA). Statistical significance was set at the 0.05 level. All *p*-values were two-sided.

## Results

### Study population

During the study period, 795,651 births were identified. Among these, we excluded deliveries from mothers who were not resident in Lombardy (*N*=125,387), who were aged less than 12 or more than 55 years at delivery (*N*=430), who had less than 22 or more than 42 weeks of gestation (*N*=1547) and who had a previous history of cancer (*N*=1687). In addition, we excluded 625 deliveries with missing values on Apgar score, 483 on vitality status and 77 on birth weight, 7408 deliveries who did not match to a hospital ICD-9 code related to childbirth and 39 deliveries with infant missing identification code. Thus, the final cohort included 657,968 deliveries.

Overall, 853 women with PAC were observed. After excluding 24 cases with non-cancer related primary diagnosis (likely not incident cases), 831 women with PAC were selected, corresponding to 1.26 cases per 1000 deliveries. Those were matched to 3324 cancer-free women. Among PAC, 30 (3.6%), 53 (6.4%) and 103 (12.4%) were diagnosed, respectively, during the first, the second and third trimester of pregnancy, while 645 (77.6%) were diagnosed during the year post-pregnancy. The most common cancer site was breast (*N*=259, corresponding to 39.3 cases per 100,000 births), followed by thyroid (*N*=138, corresponding to 21.0 cases per 100,000 births) and lymphoma (*N*=103, corresponding to 15.3 cases per 100,000 births) (See Supplementary Table S2 for a detailed list of all cancer sites).

Maternal characteristics between PAC and cancer-free women are shown in Table 1. Average maternal age was 34.8 years. No differences were observed on nationality, marital status, education and employment, type of conception and history of diabetes and hypertension. Maternal characteristics of the unmatched initial cohort are reported in Supplementary Table S3.

**Table 1:** Maternal characteristics in the matched cohort of 831 women with pregnancy associated cancer (PAC) and 3324 cancer-free women. Lombardy, Italy. 2008–2017

	Pregnancy associated cancer women *N*=831	Cancer-free women *N*=3324	Standardized difference (absolute)
	N (%)	N (%)	
Maternal Age (Year) ^a^			
< 30	107 (12.9)	428 (12.9)	Matching variable
30–34	260 (31.3)	1040 (31.3)	
35–40	379 (45.6)	1516 (45.6)	
> 40	85 (10.2)	340 (10.2)	
Mean (SD)	34.8 (4.6)	34.8 (4.6)	
Calendar Year at Birth ^a, b^			
2008	84 (10.1)	336 (10.1)	Matching variable
2009	75 (9.0)	300 (9.0)	
2010	100 (12.0)	400 (12.0)	
2011	98 (11.8)	392 (11.8)	
2012	83 (10.0)	332 (10.0)	
2013	82 (9.9)	328 (9.9)	
2014	87 (10.5)	348 (10.5)	
2015	84 (10.1)	336 (10.1)	
2016	86 (10.4)	344 (10.4)	
2017	52 (6.3)	208 (6.3)	
Nationality			
Italian	725 (87.2)	2808 (84.5)	0.078
Foreign	106 (12.8)	516 (15.5)	−0.078
Marital Status			
Married	575 (69.2)	2272 (68.4)	0.018
Not Married	234 (28.2)	981 (29.5)	−0.030
Missing	22 (2.6)	71 (2.1)	0.033
Education			
Middle School or lower	163 (19.6)	711 (21.4)	- 0.044
High School	382 (46.0)	1471 (44.3)	0.034
University or upper	282 (33.9)	1118 (33.6)	0.006
Missing	4 (0.5)	24 (0.7)	−0.031
Employment			
Employed	665 (80.0)	2616 (78.7)	0.033
Not employed	165 (19.9)	708 (21.3)	−0.036
Missing	1 (0.1)	0 (0.0)	0.049
Type of conception			
Spontaneous	799 (96.1)	3180 (95.7)	0.024
Assisted	29 (3.5)	132 (4.0)	- 0.025
Missing	3 (0.4)	12 (0.4)	0.0
History of diabetes			
No	822 (98.9)	3307 (99.5)	- 0.067
Yes	9 (1.1)	17 (0.5)	0.067
History of hypertension			
No	800 (96.3)	3233 (97.3)	- 0.057
Yes	31 (3.7)	91 (2.7)	0.057

^a^Matching variable

^b^The study cohort includes all women who delivered from 01/01/2008 to 30/06/2017

### Obstetric outcomes

PAC diagnosed during pregnancy were positively associated with induction of labor or planning of delivery (aPR=1.80, 95% CI: 1.57–2.07), cesarean section (aPR=1.78, 1.49–2.11) and premature birth (aPR=6.34, 4.59–8.75). In particular, we observed a slightly higher association between PAC diagnosed during pregnancy and preterm births fewer the 32th week, rather than those between the 28th and the 32th week and those between the 32th and the 34th week; corresponding aPR are 8.41 (2.74–25.83), 8.07 (3.31–19.64) and 7.26 (5.00–10.76) (data not shown). No association with obstetric outcomes was found among PAC diagnosed in the post-pregnancy. Corresponding aPR are 1.08 (0.98–1.20), 1.03 (0.91–1.17) and 1.18 (0.90–1.55) (Table 2).

**Table 2:** Adverse obstetric outcomes among 831 women with pregnancy associated cancer (PAC) and 3324 matched cancer-free women. Lombardy, Italy. 2008–2017

	Labor induction or planning of delivery ^a^ (Induced or elective CS Vs Spontaneous)			Mode of delivery ^b^ (Emergency or elective CS Vs vaginal or instrumental)			Timing of delivery (Very low, low, moderate or late preterm ^c^ Vs at term)		
	N (%) of events	PR (95% CI)	aPR (95% CI)	N (%) of events	PR (95% CI)	aPR (95% CI)	N (%) of events	PR (95% CI)	aPR (95% CI)
Cancer-free	299 (40.9)	1^d^	1^d^	226 (30.4)	1^d^	1^d^	48 (6.5)	1^d^	1^d^
PAC during pregnancy	139 (76.0)	1.86 (1.65–2.10)	1.80 (1.57–2.07)	100 (54.0)	1.78 (1.50–2.11)	1.78 (1.49–2.11)	77 (41.4)	6.42 (4.65–8.86)	6.34 (4.59–8.75)
Cancer-free	1027 (40.3)	1^d^	1^d^	807 (31.5)	1^d^	1^d^	198 (7.7)	1^d^	1^d^
PAC post-pregnancy	283 (44.2)	1.10 (0.99–1.21)	1.08 (0.98–1.20)	209 (32.6)	1.03 (0.91–1.15)	1.03 (0.91–1.17)	61 (9.5)	1.23 (0.94–1.62)	1.18 (0.90–1.55)

*PR* Prevalence ratio; *aPR* adjusted Prevalence Ratio; *CI* Confidence Intervals; *CS* Cesarean Section; *PAC* Pregnancy Associated Cancer

^a^Were excluded from the analysis 8 and 46 observations with missing values among PAC and non PAC women, respectively

^b^Were excluded from the analysis 4 and 16 observations with missing values among PAC and non PAC women, respectively

^c^Very low preterm birth: before 28th week, low preterm birth: between the 28th and the 32th week, moderate preterm birth: between the 32th and the 34th week, late preterm birth: between the 34th and the 37th week

^d^Reference category

Among preterm births, a significantly higher proportion of induced deliveries or elective cesarean sections emerged among PAC women (70.3% vs 46.4%, *p*-value< 0.001, data not shown).

### Neonatal outcomes

No associations between PAC and SGA newborns was observed, neither for cancer diagnosed during pregnancy (aPR=0.71, 0.36–1.35) nor for those diagnosed post-pregnancy (aPR=1.04, 0.78–1.39) (Table 3). Also comparing frequencies of SGA among women with cancer diagnosed during pregnancy and women with cancer diagnosed post-pregnancy we did not observe differences (*p*-value=0.2325). Newborns of women with PAC diagnosed during pregnancy had a higher risk of borderline significance of a low Apgar score (aPR=2.65, 0.96–7.33) as compared to cancer-free women, whereas no difference was found for women with PAC diagnosed post-pregnancy (aPR=0.66, 0.30–1.46) (Table 3). A few cases of newborn malformations (0 among PAC and 3 among cancer-free women) and perinatal mortality (1 among PAC and 2 among cancer-free women) were observed. Newborns had a lower average birth weight among PAC women with respect to cancer-free women (3109±588 g vs 3232±522 g, *p*-value< 0.0001). Further, a lower average birth weight was observed among PAC diagnosed during pregnancy, with respect to PAC diagnosed after pregnancy (2853±578 g vs 3183±570 g, *p*-value< 0.0001).

**Table 3:** Adverse neonatal outcomes among 831 women with pregnancy associated cancer (PAC) and 3324 matched cancer-free women. Lombardy, Italy. 2008–2017

	Small for gestational age (Yes Vs No)			Apgar 5th Minute (Low Vs Normal)			Malformations (Yes Vs No)			Perinatal Mortality (Stillbirth Vs Livebirth)		
	N (%) events	PR (95% CI)	aPR (95% CI)	N (%) events	PR (95% CI)	aPR (95% CI)	N (%) events	PR (95% CI)	aPR (95% CI)	N (%) events	PR (95% CI)	aPR (95% CI)
Cancer-free	57 (7.7)	1 ^a^	1 ^a^	9 (1.2)	1 ^a^	1 ^a^	1 (0.1)	1 ^a^	1 ^a^	4 (0.5)	1 ^a^	1 ^a^
PAC during pregnancy	10 (5.4)	0.70 (0.37–1.35)	0.71 (0.36–1.35)	6 (3.2)	2.67 (0.96–7.40)	2.65 (0.96–7.33)	0 (0.0)	Not estimable		1 (0.5)	1.00 (0.11–8.89)	1.01 (0.11–8.29)
Cancer-free	204 (7.9)	1 ^a^	1 ^a^	41 (1.6)	1 ^a^	1 ^a^	1 (0.0)	1 ^a^	1 ^a^	6 (0.2)	1 ^a^	1 ^a^
PAC post-pregnancy	54 (8.4)	1.06 (0.79–1.41)	1.04 (0.78–1.39)	8 (1.2)	0.78 (0.37–1.66)	0.66 (0.30–1.46)	3 (0.5)	12.00 (1.2–115.2)	11.53 (1.2–111.5)	2 (0.3)	1.33 (0.27–6.59)	0.59 (0.07–4.94)

*PR* Prevalence ratio; *aPR* adjusted Prevalence Ratio; *CI* Confidence Intervals

^a^Reference category

Among women diagnosed with PAC during pregnancy we detected 27 (14.5%) women exposed to chemotherapy. No association between adverse neonatal outcomes and exposure to chemotherapy was found, in particular for SGA newborns (*p*-value=0.6768) and low Apgar score to 5 min (p-value=0.1835). However, we observed that chemotherapy may affect the birth weight. The mean weight of birth among infants exposed to chemotherapy is lower if compared with those not exposed (respectively 2462.33 ± 464.65 g and 2919.04 ± 570.62 g, p-value=0.0001).

Some differences in selected outcomes emerged when stratifying for the most common cancer site (i.e. breast cancer, thyroid and lymphoma). With regard to PAC diagnosed during pregnancy, elective cesarean section was more common among breast and lymphoma cases than thyroid cases (respectively 82.1% vs 82.4% vs 52.9%, *p*-value = 0.048), preterm births were more frequent among breast and lymphoma cases compared to thyroid cases (respectively 52.2% vs 44.4% vs 17.6%, p-value = 0.037) and small for gestational age newborn were more frequent among thyroid cancer and lymphoma, as compared to breast cancer (respectively, 5.1%, 11.1 and 0%, *p*=0.036). No differences emerged between strata of cancer sites among PAC diagnosed after pregnancy (Table 4).

**Table 4:** Adverse obstetric and neonatal outcomes stratified by timing of diagnosis and the three most frequent cancer site. Lombardy, Italy. 2008–2017

	During Pregnancy Cancers			Post-pregnancy Cancer		
	Breast *N*=69	Thyroid *N*=17	Lymphoma *N*=18	Breast *N*=190	Thyroid *N*=121	Lymphoma *N*=85
**Obstetric outcomes**						
Labor induction or planning of delivery ^b^						
Not Induced	12 (17.9)	8 (47.1)	3 (17.6)	109 (58.0)	66 (55.0)	52 (61.2)
Induced or Elective CS	55 (82.1)	9 (52.9)	14 (82.4)	79 (42.0)	54 (45.0)	33 (38.8)
	*p* value ^a^ = 0.048			p value = 0.675		
Mode of Delivery ^c^						
Vaginal or Instrumental	31 (44.9)	12 (70.6)	6 (35.3)	129 (57.9)	88 (72.7)	58 (69.1)
CS	38 (55.1)	5 (29.4)	11 (64.7)	61 (32.1)	33 (27.3)	26 (30.9)
	p value = 0.089			p value = 0.659		
Timing of Birth						
At Term	33 (47.8)	14 (82.4)	10 (55.6)	170 (89.5)	111 (91.7)	79 (92.9)
Preterm	36 (52.2)	3 (17.6)	8 (44.4)	20 (10.5)	10 (8.3)	6 (7.1)
	p value = 0.037			p value = 0.607		
**Neonatal outcomes**						
SGA						
No	69 (100.0)	16 (94.1)	16 (88.9)	172 (90.5)	116 (95.9)	74 (87.1)
Yes	0 (0.0)	1 (5.9)	2 (11.1)	18 (9.5)	5 (4.1)	11 (12.9)
	p value ^a^ = 0.036			p value = 0.071		
Apgar 5th Minute						
Normal	66 (95.6)	17 (100.0)	18 (100.0)	188 (99.0)	120 (99.2)	85 (100.0)
Low	3 (4.4)	0 (0.0)	0 (0.0)	2 (1.0)	1 (0.8)	0 (0.0)
	p value ^a^ = 1.000			p value ^a^ = 1.000		
Malformation						
No	69 (100.0)	17 (100.0)	18 (100.0)	190 (100.0)	119 (98.4)	85 (100.0)
Yes	0 (0.0)	0 (0.0)	0 (0.0)	0 (0.0)	2 (1.6)	0 (0.0)
	–			p value ^a^ = 0.139		
Perinatal Mortality						
Livebirth	69 (100.0)	17 (100.0)	18 (100.0)	190 (100.0)	120 (99.2)	85 (100.0)
Stillbirth	0 (0.0)	0 (0.0)	0 (0.0)	0 (0.0)	1 (0.8)	0 (0.0)
	–			p value ^a^ = 0.520		

*CS* cesarean section

^a^using Fisher’s Exact Test

^b^Not including 3 missing data among the non-pregnancy cancer associated women and 3 missing data among the pregnancy cancer associated women

^c^Not including 1 missing data among the non-pregnancy cancer associated women and 1 missing data among the pregnancy cancer associated women

## Discussion

In the current study, PAC diagnosed in pregnancy was associated with an increased risk of labor induction or planned delivery, cesarean section and preterm birth. No associations between PAC and SGA newborns was observed. Nevertheless, PAC were associated to a lower birth weight. Although not significant, newborns of women with PAC diagnosed during pregnancy had a higher risk to obtain a low Apgar score at 5 min. These two adverse neonatal outcomes may be derived by the high incidence of births fewer than 37 weeks; generally premature newborns have a low weight because of their gestational age and they have a tardier adaptation to extra-uterine life after delivery. Therefore, we observed that a diagnosis of PAC had a major impact on the clinical management delivery rather than on the outcomes of newborns. Type and timing of delivery were influenced by the presence of cancer and could lead to consequences on neonatal health.

As previously observed [8–10, 20] also in our population elective cesarean section was the preferred mode of delivery among women with PAC, and iatrogenic or induced preterm birth might have been preferred to allow the beginning of cancer treatment. However, awareness is growing that cancer can be treated during pregnancy and the proportion of patients with PAC who receive antenatal treatment increases [21, 22]. Cancer treatment during pregnancy should limit iatrogenic prematurity [7, 23].

Our results are generally consistent with previous published data from record linkage studies. In a study from Australia between 1994 and 2008 [9] women with cancer diagnosed during pregnancy had higher rates of labor induction, caesarean section and planned preterm birth with respect to healthy women. The study also reported an association of PAC with multiple pregnancies and large for gestational age (LGA) newborns. Another study carried out in Denmark and Sweden between 1973 and 2008 [20] showed a positive association between PACs and adverse birth outcomes in the offspring (i.e. caesarean section birth, preterm birth, low birth weight, low Apgar score). Recent evidence from Swedish data reported also that maternal cancer during pregnancy was positively associated with an increased risk of fatal outcomes (i.e. stillbirth and infant mortality), mainly among SGA and preterm newborn [24].

Only few data are available on the potential differences of the impact on obstetric outcome in women with PAC of different sites. In our study, elective cesarean section was more common among births with a maternal lymphoma than those with a maternal breast or thyroid cancer. Abdominal-pelvic or cervical cancer is a primary indication to cesarean section [3, 23]. Moreover, the premature births were more common among women with PAC and a high rate of these were iatrogenic or induced deliveries. Spontaneous labor occurred only for the 23.7% of preterm births among women diagnosed with PAC. In our study, women with thyroid cancer did not follow this pattern, giving birth at term in the most cases.

Medical interventions aimed to anticipating birth due to cancer could cause adverse neonatal outcomes [8, 9, 20, 24]. In our study, the higher risks observed among women with PAC during pregnancy for low birth weight and low Apgar score may be driven by the increased frequency of preterm and iatrogenic birth. These findings were consistent with a large population-based study conducted in Denmark and Sweden [20].

Some studies reported an increase in the risk of SGA [20, 24]. Overall, our study did not support this finding, but we observed a higher frequency of SGA following exposure to lymphoma during pregnancy. Studies focusing on PAC and the risk of malformation in newborns generally assessed different potential unfavorable maternal exposures, such as ionizing radiation or chemotherapy [11]. Despite not considering these maternal exposures, in general, we did not observe an increased risk of malformations among PAC cases. Maternal cancer during pregnancy is also associated with an increased risk of fatal outcomes (i.e. stillbirth and infant mortality). This effect may be caused by premature births and SGA [24]. However, in our population the frequency of perinatal mortality was very low, precluding the possibility of comparison between PAC and cancer-free women.

A major strength of this study is its population-based design. HCU databases represent large and validated sources of data that cover patient information over a span of 10 years. HCU databases collect hospitalizations of all Lombardy hospitals and the linkage to CedAP database allowed to assess detailed information about each delivery identified, including a wide range of perinatal outcomes.

Some weaknesses warrant consideration. We defined cancer diagnosis as the first hospital admission due to malignant disease. However, if the first diagnosis was performed at an outpatient setting, hospital data may be less efficient in identifying exact diagnosis timing. Considering all cancer sites overall might be a limitation of our study, as different cancers could have different course and implications on pregnancy outcomes. Moreover, according to our primary aim of assessing the association between PAC and adverse perinatal outcomes, only deliveries (not all pregnancy) were included in the study. The information about the outcomes of interest were collected from CedAP which is filled in only if a delivery is completed.

## Conclusions

In our study, PAC was associated with iatrogenic preterm birth. Consequentially, among PAC women birth weight was lower compared to non PAC women and an increased risk of low Apgar score at 5 min emerged (although non statistically significant). The high incidence of iatrogenic and preterm birth may justify these findings, in fact premature newborns have a lower weight and they obtain a lower Apgar score if compared with at term newborns. In order to guarantee neonatal wellbeing in offspring of women with PAC, careful decision-making strategy regarding mode and timing of delivery should be considered. Maternal and fetal health allowing iatrogenic preterm birth should be limited.

## Supplementary Information

**Additional file 1.**

**Additional file 2.**

**Additional file 3.**

## Data Availability

The data that support the findings of this study are available from Lombardy Region, but restrictions apply to the availability of these data which were used under license for the current study, and so are not publicly available. Data are however available from the authors upon reasonable request and with permission of Lombardy Region.

## Abbreviations

aPR: Adjusted PrevalenceRatio
ATC: Anatomical Therapeutic Chemical
CeDAP: Certificates of Delivery Assistance
CI: Confidence Interval
FCS: Fully Conditional Specification
GEE: Generalized Estimating Equations
HCU: HealthCare Utilization
ICD: International Classification of Diseases
IUGR: Intrauterine Growth Restriction
NHS: National Health System
OR: Odds Ratio
PAC: Pregnancy associated cancer
PPROM: Premature Membrane Rupture
PR: Prevalence Ratio
SGA: Small for Gestational Age
