# Supplementary material for: Obstetric and neonatal outcomes in women with pregnancy associated cancer: a population-based study in Lombardy, Northern Italy
Source: BMC Pregnancy Childbirth. 2021 Jan 7;21:31. doi: 10.1186/s12884-020-03508-4 (PMC7791735; doi:10.1186/s12884-020-03508-4)
Supplement: Supplementary file 1 — Additional file 1. [file 12884_2020_3508_MOESM1_ESM.docx]

**Table S1.** Code list of diseases/conditions and medicaments drugs used for the current study.

| **Disease/condition** | **DRG codes** |
| --- | --- |
| Delivery | 370-375 |
| **Disease/condition** | **ICD9-CM codes** |
| Any malignant cancer | 140-208 |
| Specific cancer sites | Head and neck: 140-149, 160; other gastrointestinal tract: 150-152, 156, 158, 159; colorectum: 153, 154, liver: 155; pancreas: 157; lung and other respiratory tract: 161-163, 165; skeletal/connective tissue: 170, 171; melanoma 172; skin, excluding melanoma: 173; breast: 174; other gynecological: 179, 182, 184; cervix: 180; placenta: 181; ovary:183; kidney and urinary tract: 188, 189.0-189.4, 189.8-189.9; nervous system: 191, 192; thyroid: 193; lymphoma 196, 200-202; multiple myeloma: 203; leukemia: 204-208; other site: 164, 190, 194. |
| **Drug** | **ATC codes** |
| Hypertension | C02, C03, C07, C08, C09 |
| Type 2 diabetes | A10 |
