# Supplementary material for: Obstetric and neonatal outcomes in women with pregnancy associated cancer: a population-based study in Lombardy, Northern Italy
Source: BMC Pregnancy Childbirth. 2021 Jan 7;21:31. doi: 10.1186/s12884-020-03508-4 (PMC7791735; doi:10.1186/s12884-020-03508-4)
Supplement: Supplementary file 2 — Additional file 2. [file 12884_2020_3508_MOESM2_ESM.docx]

**Table S2.** Classification of pregnancy associated cancer by site of diagnosis according to the timing of diagnosis among 657,968 deliveries. Lombardy, Italy. 2008-2017.

|  |  | During Pregnancy Cancers  (N=186) | |  | Post-pregnancy cancers  (N=645) | |  | All cancers  (N=831) | |
| --- | --- | --- | --- | --- | --- | --- | --- | --- | --- |
| Cancer site |  | N | Risk^a^ |  | N | Risk^a^ |  | N | Risk^a^ |
| Breast |  | 69 | 10.48 |  | 190 | 28.86 |  | 259 | 39.34 |
| Thyroid |  | 17 | 2.58 |  | 121 | 18.38 |  | 138 | 20.96 |
| Lymphoma^b^ |  | 18 | 2.73 |  | 85 | 12.91 |  | 103 | 15.28 |
| Cervix |  | 17 | 2.58 |  | 35 | 5.32 |  | 52 | 7.90 |
| Skin, excluding melanoma |  | 11 | 1.67 |  | 35 | 5.32 |  | 46 | 6.99 |
| Colorectum |  | 4 | 0.61 |  | 24 | 3.65 |  | 28 | 4.26 |
| Kidney and urinary tract |  | 5 | 0.76 |  | 21 | 3.19 |  | 26 | 3.95 |
| Nervous system |  | 4 | 0.61 |  | 21 | 3.19 |  | 25 | 3.80 |
| Melanoma |  | 9 | 1.37 |  | 14 | 2.13 |  | 23 | 3.50 |
| Ovary |  | 8 | 1.22 |  | 11 | 1.67 |  | 19 | 2.88 |
| Skeletal/connective tissue |  | 2 | 0.30 |  | 17 | 2.58 |  | 19 | 2.88 |
| Head and neck |  | 7 | 1.06 |  | 11 | 1.67 |  | 18 | 2.73 |
| Lung and other respiratory tract |  | 1 | 0.15 |  | 17 | 2.58 |  | 18 | 2.73 |
| Leukemia |  | 6 | 0.91 |  | 9 | 1.37 |  | 15 | 2.28 |
| Other gastrointestinal tract |  | 2 | 0.30 |  | 9 | 1.37 |  | 11 | 1.67 |
| Pancreas |  | 1 | 0.15 |  | 5 | 0.76 |  | 6 | 0.91 |
| Placenta |  | 0 | 0.00 |  | 4 | 0.61 |  | 4 | 0.61 |
| Other gynecological tract |  | 1 | 0.15 |  | 3 | 0.46 |  | 4 | 0.61 |
| Liver |  | 1 | 0.15 |  | 2 | 0.30 |  | 3 | 0.46 |
| Multiple myeloma |  | 0 | 0.00 |  | 1 | 0.15 |  | 1 | 0.15 |
| Other sites |  | 2 | 0.30 |  | 5 | 0.76 |  | 7 | 1.06 |
| Not defined |  | 1 | 0.15 |  | 5 | 0.76 |  | 6 | 0.91 |
| All cancers |  | 186 | 28.25 |  | 645 | 97.97 |  | 831 | 126.22 |
| ^a^ Risk of pregnancy-associated cancer x 100,000 pregnancies.  ^b^ 21 observations are secondary lymph node carcinoma | | | | | | | | | |
