# Supplementary material for: Obstetric and neonatal outcomes in women with pregnancy associated cancer: a population-based study in Lombardy, Northern Italy
Source: BMC Pregnancy Childbirth. 2021 Jan 7;21:31. doi: 10.1186/s12884-020-03508-4 (PMC7791735; doi:10.1186/s12884-020-03508-4)
Supplement: Supplementary file 3 — Additional file 3. [file 12884_2020_3508_MOESM3_ESM.docx]

**Table S3.** Maternal Characteristics in the original cohort of 831 women with pregnancy associated cancer (PAC) and 657,137 cancer-free women. Lombardy, Italy. 2008-2017.

|  | Pregnancy associated cancer women  N=831 | Cancer-free women  N=657,137 | Standardized difference  (absolute) | |
| --- | --- | --- | --- | --- |
|  | N (%) | N (%) |  | |
| Maternal Age (Year) |  |  |  | |
| < 30 | 107 (12.9) | 168,841 (25.7) | - 0.329 | |
| 30-34 | 260 (31.3) | 239,686 (36.5) | - 0.110 | |
| 35-40 | 379 (45.6) | 214,575 (32.7) | 0.267 | |
| >40 | 85 (10.2) | 34,035 (5.2) | 0.188 | |
| Mean (SD) | 34.8 (4.6) | 32.7 (5.1) |  | |
| Calendar Year at Birth ^a^ |  |  |  | |
| 2008 | 84 (10.1) | 71,141 (10.8) | - 0.023 | |
| 2009 | 75 (9.0) | 70,379 (10.7) | - 0.057 | |
| 2010 | 100 (12.0) | 68,591 (10.4) | 0.051 | |
| 2011 | 98 (11.8) | 72,356 (11.0) | 0.025 | |
| 2012 | 83 (10.0) | 71,703 (10.9) | - 0.029 | |
| 2013 | 82 (9.9) | 69,504 (10.6) | - 0.023 | |
| 2014 | 87 (10.5) | 69,049 (10.5) | 0.000 | |
| 2015 | 84 (10.1) | 67,582(10.3) | - 0.007 | |
| 2016 | 86 (10.4) | 66,023 (10.1) | 0.010 | |
| 2017 | 52 (6.3) | 30,809 (4.7) | 0.070 | |
| Nationality |  |  |  | |
| Italian | 725 (87.2) | 539,236 (82.1) | 0.142 | |
| Foreign | 106 (12.8) | 117,901 (17.9) | - 0.142 | |
| Marital Status |  |  |  | |
| Married | 575 (69.2) | 438,434 (66.7) | 0.053 | |
| Not Married | 234 (28.2) | 203,714 (31.0) | -0.062 | |
| Missing | 22 (2.6) | 14,986 (2.3) | 0.024 | |
| Educational |  |  |  | |
| Middle School or lower | 163 (19.6) | 160,078 (24.4) | -0.115 | |
| High School | 382 (46.0) | 298,175 (45.4) | 0.012 | |
| University or upper | 282 (33.9) | 194,776 (29.6) | 0.092 | |
| Missing | 4 (0.5) | 4108 (0.6) | -0.019 | |
| Employment |  |  |  | |
| Employed | 665 (80.0) | 493,564 (75.1) | 0.118 | |
| Not employed | 165 (19.9) | 163,537 (24.8) | -0.121 | |
| Missing | 1 (0.1) | 36 (0.1) | 0.046 | |
| Type of conception |  |  |  | |
| Spontaneous | 799 (96.1) | 636,064 (96.8) | -0.035 | |
| Assisted | 29 (3.5) | 17,848 (2.7) | 0.045 | |
| Missing | 3 (0.4) | 3225 (0.5) | -0.020 | |
| History of diabetes |  |  |  | |
| No | 822 (98.9) | 653,375 (99.4) | - 0.054 | |
| Yes | 9 (1.1) | 3,762 (0.6) | 0.054 | |
| History of hypertension |  |  |  | |
| No | 800 (96.3) | 640,344 (97.4) | - 0.063 | |
| Yes | 31 (3.7) | 16,793 (2.6) | 0.063 | |
| ^a^ Were included in the study cohort all women who delivered from 01/01/2008 to 30/06/2017 | | | |  |
